# Rat Liver Enzyme Release Depends on Blood Flow-Bearing Physical Forces Acting in Endothelium Glycocalyx rather than on Liver Damage

**DOI:** 10.1155/2017/1360565

**Published:** 2017-02-28

**Authors:** Julieta A. Díaz-Juárez, Rolando Hernández-Muñoz

**Affiliations:** ^1^División de Ciencias Biológicas y de la Salud, Departamento de Atención a la Salud, Universidad Autónoma Metropolitana (UAM Xochimilco), 04960 Ciudad de México, Mexico; ^2^Departamento de Biología Celular y Desarrollo, Instituto de Fisiología Celular, Universidad Nacional Autónoma de México (UNAM), 04510 Ciudad de México, Mexico

## Abstract

We have found selective elevation of serum enzyme activities in rats subjected to partial hepatectomy (PH), apparently controlled by hemodynamic flow-bearing physical forces. Here, we assess the involvement of stretch-sensitive calcium channels and calcium mobilization in isolated livers, after chemical modifications of the endothelial glycocalyx and changing perfusion directionality. Inhibiting in vivo protein synthesis, we found that liver enzyme release is influenced by de novo synthesis of endothelial glycocalyx components, and released enzymes are confined into a liver “pool.” Moreover, liver enzyme release depended on extracellular calcium entry possibly mediated by stretch-sensitive calcium channels, and this endothelial-mediated mechanotransduction in liver enzyme release was also evidenced by modifying the glycocalyx carbohydrate components, directionality of perfusing flow rate, and the participation of nitric oxide (NO) and malondialdehyde (MDA), leading to modifications in the intracellular distribution of these enzymes mainly as nuclear enrichment of “mitochondrial” enzymes. In conclusion, the flow-induced shear stress may provide fine-tuned control of released hepatic enzymes through mediation by the endothelium glycocalyx, which provides evidence of a biological role of the enzyme release rather to be merely a biomarker for evaluating hepatotoxicity and liver damage, actually positively influencing progression of liver regeneration in mammals.

## 1. Introduction

Increased plasma enzyme activities are considered as diagnostic features for several diseases [1], since the release of enzymes usually follows their respective concentration gradients between an organ, such as the liver, and the blood compartments [2]. In fact, values of released enzymes are much higher than the apparent disappearance rate constants after acute liver injury [3]. Moreover, aspartate aminotransferase (AST), as part of a physiological model for end-stage liver disease (MELD) score, can be useful for pretransplant graft allocation, as well as for postoperative risk stratification [4].

Numerous enzymes are produced in the liver and are normally distributed within the cells of the liver [5], and elevation of serum enzyme is taken as a sensitive biomarker of liver toxicity. For instance, elevated transaminases level in conjunction with a rise in bilirubin level to more than the double is considered as a marker index of hepatotoxicity [6]. In the case of nonalcoholic fatty liver disease (NAFLD), high levels of serum cholesterol are associated with a resultant liver injury characterized by hepatomegaly and accompanied by increased activities of AST and aspartate aminotransferase (ALT) enzymes [7]. On the other hand, alcoholic subjects, having moderate/severe hepatic steatosis, usually present an increase in the levels of triglycerides, cholesterol, glucose, *γ*-glutamyl transpeptidase (*γ*-GTP), ALT, and bilirubin and a decrease in the levels of AST [8]. Indeed, it has been considered that the AST/ALT ratio could be a marker playing a role in alcoholic liver disease progression [8]. However, there exist discrepancies when matching changes of assumed “liver enzymes” in serum and other markers for liver integrity. For instance, increased levels of *γ*-GTP, a liver enzyme, play an independent role in the pathogenesis and clinical evolution of cardiovascular disease induced by atherosclerosis, and there is an association of increased ALT levels and cardiovascular disease [9]. Therefore, several situations arise where there is an evident loss of correlation between serum levels of liver enzymes and tissue necrosis, as well as in the specificity of possible tissue markers. The latter can challenge the truly diagnostic usefulness based on determinations of serum enzyme activities.

Indeed, we can say that the control of cellular enzyme release remains poorly understood. In fact, we think that there are at least two issues that have not received appropriate attention: besides their usefulness as diagnostic tools, what is the physiological role, if any, of serum enzymes activities? Second, what are the mechanisms governing the enzyme release by a determined tissue? Regarding the second question, we demonstrated that an important fraction of the released hepatic enzymes depends largely on hemodynamic changes in the rat liver [10]. Taking advantage of the model of two-thirds partial hepatectomy- (PH-) induced rat liver regeneration, we showed that liver cell proliferation occurs accompanied by a selective elevation of serum enzymes, not related to hepatocellular necrosis [11], which is partly regulated by flow-bearing physical forces, independently of extrahepatic factors [10].

The mechanical environment of mammalian cells is defined by complex interactions between gravitational forces and intracellular tension within the cytoskeletal elements [12]. The endothelium is located between the flowing wall and the vascular arterial wall and is exposed to shear stress [12], which activates many signal transduction pathways in these cells [13]. Cell cytoskeleton generates physical forces transmitted throughout the cells, integrating a system of mechanochemical signal transduction, enabling cells to perceive and respond to physical stress, such as shear stress [14], which activate various pathways and regulate expression of extracellular matrix components [15].

This so named mechanotransduction regulates many physiological processes in mammals, including blood pressure regulation and stretch-evoked responses in several visceral and vascular organs [16]. Indeed, a major challenge is the identification of the mechanism by which mechanical forces are converted into a sequence of intracellular biochemical signals in endothelial cells [17]. The force transduction could regulate kidney blood flow and blood pressure in the vasculature, among others, involving many molecules that have been proven difficult to identify, possibly due to their rarity [18]. Hence, the physical-chemical interactions within the cells are becoming a fascinating field of study, and the enzyme release by vascular organs can be an event regulated by hemodynamic forces.

A number of intracellular events triggered by fluid shear stress include direct stimulation of luminal surface transmembrane proteins, activation of ion channels, intracellular calcium mobilization, and production of nitric oxide (NO) [19]. This allows transduction of stress along cytoskeletal elements, and changes on endothelial cell membrane act as primary mechanoreceptors in response to shear stress [20]. A possible role for cell-mediated mechanotransduction in liver enzyme release was suggested by increasing flow-induced shear stress, which is largely controlled through modifications in cell calcium mobilization and production of liver NO [21]. Stretching forces increase intracellular calcium concentration through the stretch-sensitive channels, forming reactive oxygen species (ROS) that could be responsible for cell injury [22]. However, previous studies have suggested that applying mechanical force to integrins can activate mechanosensitive ion channels, which participate in the modulation of cytoskeleton structure or contractility [23]. Laminar shear stress exerts antiapoptotic, antiatherosclerotic, and antithrombotic effects on endothelial cells, and mechanical stretch of cardiac myocytes can modulate growth, apoptosis, electric remodelling, alterations in gene expression, and autocrine and paracrine effects [24].

Based on the aforementioned, it is possible that a fraction of serum enzyme activities largely depends on blood flow-induced mechanobiological features that control, at least partly, the rate of released hepatic enzymes. Therefore, the present study is an attempt to demonstrate this statement, by inhibiting protein synthesis in vivo, as well as by blocking participation of cell calcium and stretch-sensitive channels, chemical modifications of endothelial glycocalyx, and changes in perfusion directionality, in vitro experiments using isolated perfused livers obtained from either sham-operated rats or rats subjected to 70% PH.

## 2. Methods

### 2.1. Materials

Enzymes, coenzymes, diagnostic kits for serum enzyme activities, and other reagents were obtained from Sigma Chemical Co. (St. Louis, MO).

### 2.2. Animals and Treatments

Male Wistar rats (120 animals with 230–280 g body weight) were fed ad libitum and maintained under a 12 h light/dark period. Surgical procedures were done between 9 and 10 a.m. Sham-operated (laparotomy) animals provided a control for surgical conditions and other animals were subjected to two-thirds PH [25]. Animals were euthanized by decapitation under sodium pentobarbital anaesthesia, and liver and blood samples were collected. All procedures were done in accordance with the Mexican Federal Regulations for Animal Care and Experimentation (Ministry of Agriculture,* SAGARPA*, NOM-062-ZOO-1999).

### 2.3. Enzyme Activities in Serum and Perfusates

The following enzyme activities were quantified: lactate (LDH; EC 1.1.1.27) and glutamic (GDH; EC 1.4.1.3) dehydrogenases, alanine (ALT; EC 2.6.1.2) and aspartic (AST; EC 2.6.1.1) aminotransferases, and malate dehydrogenase (MDH; EC 1.1.3.25), as well as that of ornithine carbamoyl transferase (OCT; EC 2.1.3.3) by the methods described elsewhere [10, 11]. In addition, the levels of NO and that of malondialdehyde (MDA) were also measured in the perfusates through methods previously described elsewhere [10, 21].

### 2.4. Liver Perfusion and Changes in Flow Rate

Liver perfusion was done essentially as described by Habib et al. [26]. The perfusion solution, consisting of calcium-free Krebs-Ringer-bicarbonate-buffered saline with 5 mmols·L^−1^ glucose (pH 7.4), was oxygenated and heated at 37°C. Livers were always perfused in the physiological antegrade direction (portal to cava veins). After removing the remaining blood, the flow rate was adjusted to 2 mL·min^−1^·g^−1^, considered as the normal hepatic blood flow [21]. The perfusate solution was collected for a 15–20 min interval; thereafter, the flow rate was increased from 2 to 6 mL·min^−1^·g^−1^ of liver, and the flow rate was adjusted according to the remaining liver weight after PH [10, 21]. Here, the estimated shear stress ranged from 0.014 to 0.82 Pa (0.14–8.2 dyn/cm2), when flow rate increased from 1 to 6 mL·min^−1^·g^−1^ of liver. Since gadolinium acts as an acid at physiological pH, we used low millimolar concentrations of this lanthanide (10 mM) at acidic pH (5.2) and further dilution for applying this into the perfusion medium. Other experimental protocols were performed with the perfused liver model to test the role of 2 mmol/L calcium added to the perfusion buffered solution, as well as the addition of modifiers of the endothelium glycocalyx, such as hyaluronidase type IV (EC 3.2.1.35), heparanase III (E.C.4.2.2.8), and chondroitinase (EC 4.2.2.4) from* Proteus vulgaris*, which were dissolved in the perfusion buffered solution to a final concentration of 15 mU/mL; moreover, heat inactivation of these enzymes was performed by immersing the hyaluronidase or heparanase stock solution in boiling water for 30 min. In addition, the lectin concanavalin A bound to ferritin (MW 500 kD) was also used. The substances were injected after the baseline period, through a side branch of the inflow cannula directly into the perfusion medium for a period of 10 min in a nonrecycling fashion; under these conditions, the final concentration of ferritin-concanavalin A was 100 *μ*g/mL. All the procedures were done according to the protocol described by Suarez and Rubio [27].

### 2.5. Statistical Analysis

The rate of enzyme release into the perfusates from isolated liver was calculated by means of the kinetics Enzfitter Program, based on the consideration of the simultaneity of enzyme release observed in isolated liver preparations [28]. Significance of the differences between groups was analysed by two-way ANOVA and, in case of significance, Student's *t*-test was applied.

## 3. Results

### 3.1. Effects of Cycloheximide- (CHM-) Induced Inhibition of Protein Synthesis on PH-Promoted Enzyme Release In Vivo and In Vitro

**Figure 1:**
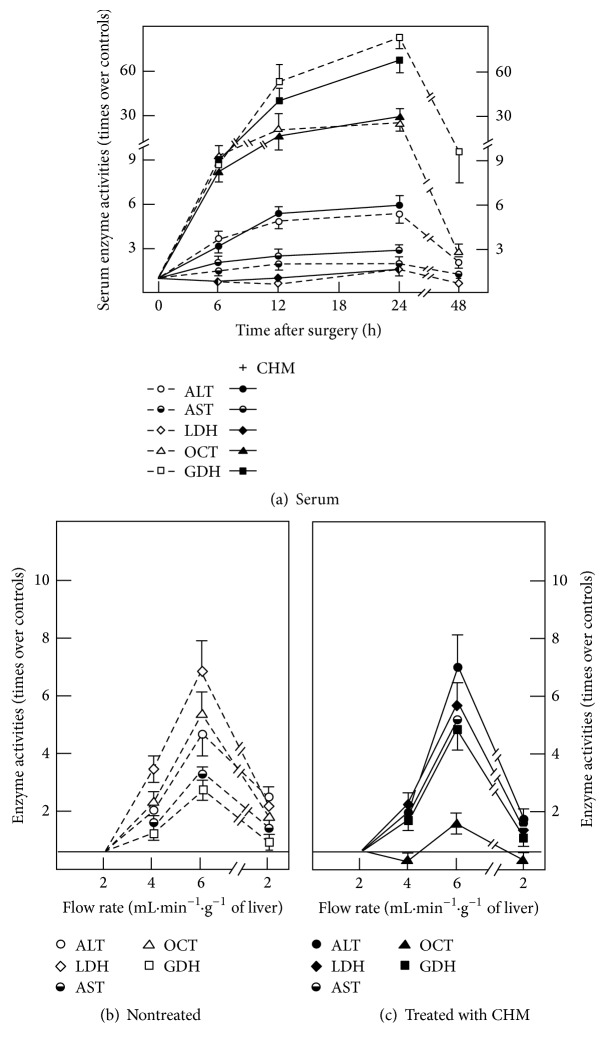
Effect of CHM administration on the pattern of serum enzyme activities at various times after 70% PH. Enzyme activities are expressed as mean ± SE for six independent determinations per experimental point for LDH, ALT, AST, GDH, and OCT, after 24 h after surgery. In panel (a), empty symbols represent 70%-PH rats, and solid symbols represent those PH animals in vivo treated with 1.8 mg/kg, b.w., of CHM, 6 h before euthanasia. Enzyme release in vitro from isolated livers from PH rats without (panel (b)) or with (panel (c)) in vivo treatment with CHM is also shown.

The profile of serum enzymes activities induced by PH was not significantly modified by CHM-induced inhibition of protein synthesis (Figure 1(a)), but CHM did change liver enzyme release as a function of increasing in vitro the flow rate in perfused organs obtained from PH animals (Figures 1(b) and 1(c)). Indeed, treatment with CHM to PH rats significantly diminished the release of OCT, whereas it enhanced that of GDH. Only under these conditions was the proportional release of GDH higher than that of OCT (Figures 1(b) and 1(c)).

### 3.2. Effects of PH on Subcellular Distribution of Hepatic Enzyme Activities

The lack of effect of inhibition of protein synthesis on liver enzyme release would suggest the existence of an “enzyme pool” for its extracellular delivery. Therefore, we determined enzyme activities in subcellular fractions obtained from livers of the experimental groups. In the cytosolic fraction, PH induced a significant decrease in LDH activity, a cytoplasmic enzyme, during the first 24 h after surgery; in addition, the activity of OCT, a mitochondrial enzyme, also was diminished in the cytosol. On the contrary, mitochondrial LDH activity was drastically increased at earlier times after surgery and remained significantly higher during all times tested. Moreover, PH also augmented the proportional activities found for aminotransferases (ALT and AST) at 12 h after surgery (Table 1) in the mitochondrial fraction; in view of the small percentage of both LDH and ALT activities in the mitochondria, their PH-induced increases in this subcellular fraction resulted to be more relevant. The PH promoted an early decrease (12 h) in microsomal GDH activity, while activities of both LDH and OCT were increased at 24 h after surgery (Table 1). The plasma membranes relative distribution of enzyme activities was also modified by PH. LDH activity increased at 72 h after surgery, while that of ALT showed two peaks of elevation (Table 1). Oppositely, both AST and OCT activities were decreased in plasma membranes. Nuclear proportional activity of these enzymes was very low. However, PH induced increased nuclear ALT and GDH activities, and that of OCT increased up to 8 times (Table 1).

### 3.3. Effects of Modifying the Endothelial Cell Membrane Glycocalyx on Enzyme Release by Perfused Livers from PH Rats

**Figure 2:**
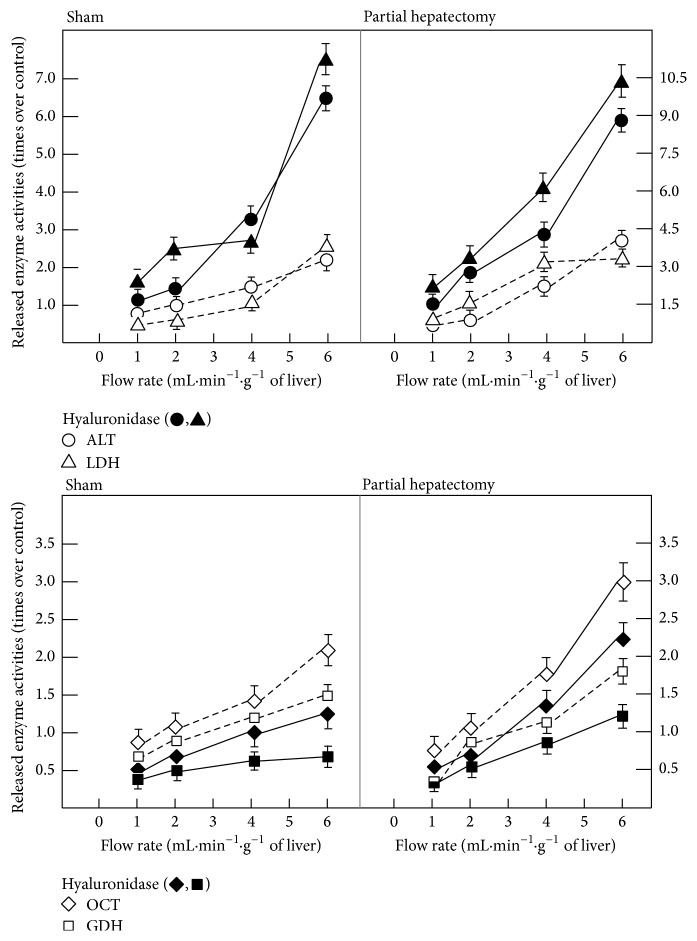
Effects of calcium and addition of hyaluronidase on endothelium glycocalyx and enzyme release by perfused isolated livers from PH rats. Results of enzyme activities are expressed as mean ± SE (six independent determinations per experimental point) of times over sham-operated (control) values for LDH, ALT, GDH, and OCT, after 24 h after surgery. To study the effect of hyaluronidase, this compound was injected via a side branch of the inflow cannula at a final concentration of 1.5 mU/mL.

**Figure 3:**
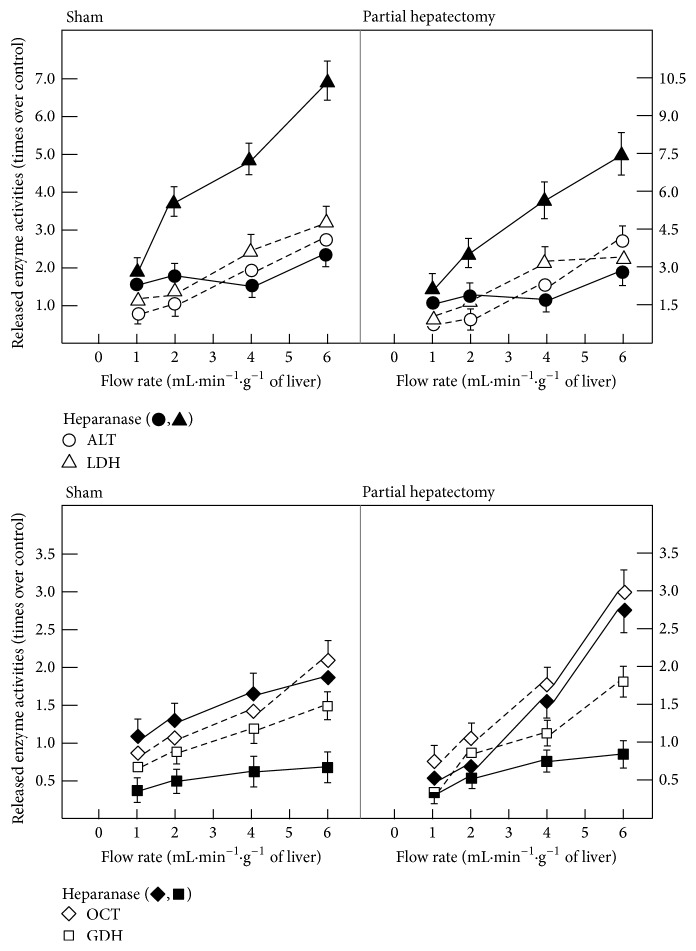
Effects of calcium and addition of heparanase on endothelium glycocalyx and enzyme release by perfused isolated livers from PH rats. Results of enzyme activities are expressed as mean ± SE (six independent determinations per experimental point) of times over sham-operated (control) values for LDH, ALT, GDH, and OCT, after 24 h after surgery. To study the effect of heparanase III, this compound was injected via a side branch of the inflow cannula at a final concentration of 1.5 mU/mL.

**Figure 4:**
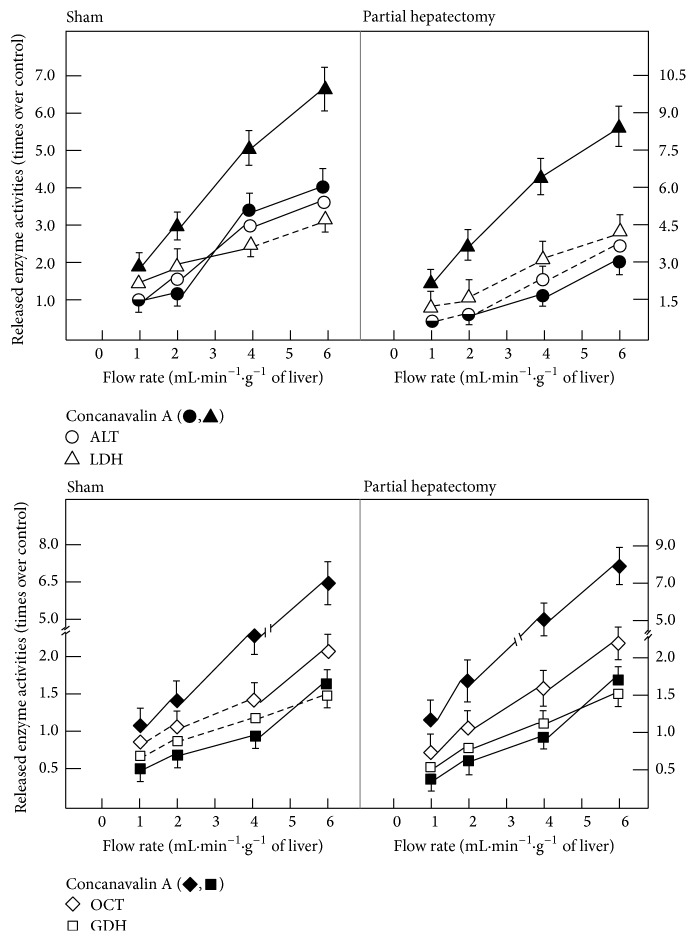
Effects of calcium and addition of concanavalin A on endothelium glycocalyx and enzyme release by perfused isolated livers from PH rats. Results of enzyme activities are expressed as mean ± SE (six independent determinations per experimental point) of times over sham-operated (control) values for LDH, ALT, GDH, and OCT, after 24 h after surgery. To study the effect of ferritin-concanavalin A complex, this compound was injected via a side branch of the inflow cannula at a final concentration of 10 *µ*g/mL.

On the other hand, the CHM-induced changes in hepatic enzyme release could be due to de novo synthesis of proteins involved in the endothelial-mediated mechanotransduction events. Hence, we conducted experiments to modify the endothelial glycocalyx (Figures 2–4). Chondroitinase however had no significant effect on liver enzyme release (not shown); hyaluronidase, heparanase, and ferritin-concanavalin A complex elicited significant effects (Figures 2–4). In control livers, the addition of hyaluronidase induced increased release of cytoplasmic enzymes such as LDH and that of ALT, while diminishing the release of mitochondrial OCT and GDH; however, the response of enzymes to increasing flow rate was conserved (Figure 2). Similar effects were found in isolated livers from PH rats, since the release of the LDH and ALT enzymes was drastically enhanced, also in response to increasing rate of perfusion flow, while that of OCT was indeed decreased under the same experimental conditions (Figure 2). With heparanase III, a different pattern was obtained (Figure 3); heparanase also increased the LDH release in response to changing the flux rate, in both control and livers from PH rats but, in a different manner than hyaluronidase, the release of ALT was not affected. Moreover, heparanase only diminished GDH release in both control and livers from PH rats (Figure 3). On the other hand, the ferritin-concanavalin A complex, which blocks glucose and mannose residues in the glycoproteins, stimulated significantly the release of LDH and OCT, while that of ALT or GDH was unaffected (Figure 4). Inactivated hyaluronidase or heparanase had no effect on the release of the enzymes tested. Altogether, this indicates that modifications of glycoproteins constituting the endothelial glycocalyx clearly affected differentially those “cytoplasmic” and “mitochondrial” enzymes, in the magnitude of response to the flow rate, while maintaining the pattern of release for each enzyme.

### 3.4. Effects of Modifying the Endothelial Cell Membrane Glycocalyx on the Production of NO and MDA by Perfused Livers from PH Rats

Since it has been previously reported that enzymatic removal of glycocalyx-contained heparan sulfates and hyaluronic acid significantly reduced the pressure induced and decreases mechanotransduction-induced increases in NO/ROS production and increased cell membrane permeability [20, 29, 30], we determined the production of both NO and MDA under our experimental conditions (Table 2). In sham-operated rats, by increasing flow rate of the liver perfusion (from 2 to 6 mL·min^−1^·g^−1^ of liver), augmented production of NO was noted. As expected, addition of hyaluronidase, heparanase, or concanavalin A did decrease significantly NO production, especially at larger flow pressures (Table 2). The PH promoted drastic production of NO, which reached a peak at flow rate of 4 mL·min^−1^·g^−1^ of liver, declining thereafter [21]. Here, the effects of glycocalyx modifiers were more marked and evident at the higher flow pressures (Table 2). In control animals, increasing rate of the perfusion flow also augmented liver production and release of MDA. In these preparations, addition of heparanase and concanavalin A, but not of hyaluronidase, decreased MDA content from perfusates obtained at the highest flow rate (Table 2). In the case of liver samples obtained from PH animals, basal MDA production was significantly higher than that of controls (Table 2). Moreover, there were significant inhibitory effects of the three glycocalyx modifiers on MDA production, noticed in the interval of flow pressures elicited by flowing rates from 4 to 6 mL·min^−1^·g^−1^ of liver (Table 2).

### 3.5. Effects of Calcium and of Inhibiting Stretch-Sensitive Ion Channels (Mechanoreceptors) by Using Gadolinium Chloride on Hepatic Enzyme Release

**Figure 5:**
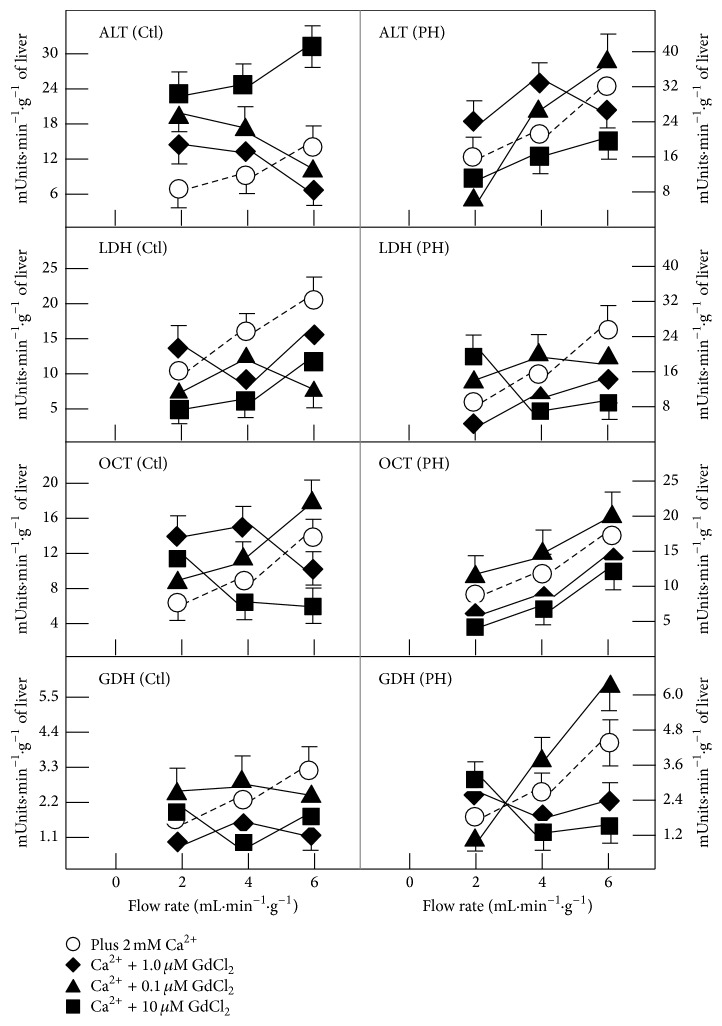
Effects of calcium and of gadolinium (inhibitor of stretch-sensitive channels) on enzyme release by livers from control and PH rats after increasing the perfusion rate. Results of enzyme activities are expressed as mean ± SE (six independent determinations per experimental point) as mUnits·min^−1^·g^−1^ of liver for LDH, ALT, GDH, and OCT. Additions to the perfusion medium were 1.8 mmol/L calcium and gadolinium chloride (0.1 to 10 *μ*moles/L). Symbols for each experimental condition are indicated at the top of the figure.

Calcium addition increased significantly ALT and LDH activities; in contrast, release of OCT activity was significantly decreased by calcium and that of GDH was also diminished, but to a lesser extent (not shown). When increasing gadolinium concentration (0.1 to 10 *μ*M) in control livers, ALT release was decreased and did not respond adequately to increasing flow rate, but with the highest gadolinium concentration (10 *μ*M) ALT responded again to the increased shear stress, but in higher magnitude (Figure 5). As to LDH, most gadolinium concentrations decreased the release for this enzyme. Regarding the mitochondrial enzymes OCT and GDH release, gadolinium chloride did enhance OCT release at lower concentrations, whereas 10 *μ*M indeed gradually decreased OCT release (Figure 5). The GDH release was greatly affected by gadolinium, since the release of this enzyme in function of flow rate was lost by the addition of the lanthanide (Figure 5).

As shown in the previous figures, hepatic enzyme release in response to the imposed flow rate is significantly increased in liver from PH rats, when compared with control sham-operated rats. In this case, the effect of gadolinium was quite different in liver preparations of animals subjected to PH. For instance, only 10 *μ*M gadolinium decreased ALT release, while a stronger effect was noted in the release for LDH, since all the concentrations of gadolinium employed abolished the LDH release in response to increasing flow rate in PH rats (Figure 5). With the mitochondrial enzymes, gadolinium did not have any significant effect on OCT release, whereas it increased that of GDH at the lowest gadolinium concentration; nonetheless, larger concentrations of the lanthanide also blocked the release of GDH in response to increasing flow rate in PH animals (Figure 5).

### 3.6. Effects of Calcium and Calcium Channel Blockers on Enzyme Release by Perfused Liver and Liver Slices from PH Rats

**Figure 6:**
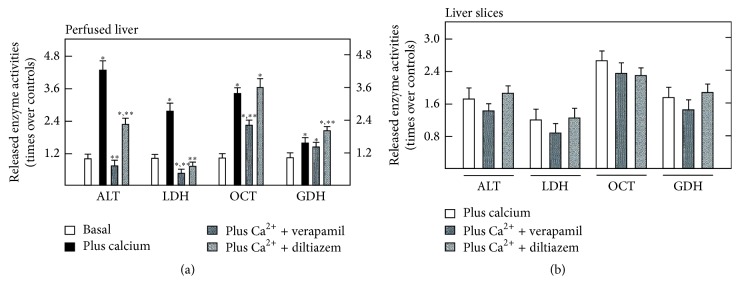
Effects of calcium and of antagonists of calcium channels on enzyme release by perfused isolated livers and liver slices from PH rats. Results of enzyme activities are expressed as mean ± SE (six independent determinations per experimental point) of times over liver preparations under basal conditions (no added calcium and a flow rate of 2 mL·min^−1^·g^−1^ of liver). Basal values for released liver enzymes were for LDH, ALT, GDH, and OCT = 7, 5, 1.2, and 4.1 mU·min^−1^·g^−1^ of liver, respectively. The additions to the perfusion medium were 2 mmol/L calcium, 30 *μ*moles/L verapamil, or 15 *μ*moles/L diltiazem, and the flow rate increased to 6 mL·min^−1^·g^−1^ of liver. At the bottom, liver slices were subjected to the same conditions. Experimental groups are as indicated at the top of the figure. Statistical significance: ^*∗*^*p* < 0.01 against the basal condition (without calcium) and ^*∗∗*^*p* < 0.01 versus enzyme release under increased flow rate in the presence of 1.8 mmoles/L calcium.

To further investigate the role of calcium as a transducer in the flow rate-bearing release of liver enzymes, inhibition of calcium entry through cell calcium channels was performed in perfused livers from PH rats subjected to changes in the rate of perfusion. Moreover, a similar approach was achieved in liver slices from the same animals, where the effect of mechanotransduction forces was minimized (Figure 6). In this case, adding calcium and increasing the flow rate (from 2 to 6 mL·min^−1^·g^−1^ of liver), the release of ALT and LDH was increased in response to the added calcium; OCT and GDH release did not respond to calcium, but it did when increasing the rate of perfusion flow (Figure 6). Moreover, under these experimental conditions (2 mmoles/L calcium and a flow rate of 6 mL·min^−1^·g^−1^ of liver), the release of both ALT and LDH was largely reduced by calcium channel blockers (verapamil and diltiazem). In fact, calcium channel blockers abrogated the effect of the perfusion rate on ALT release, with verapamil being a stronger inhibitor (Figure 6(a)). In a similar fashion, LDH release was readily inhibited by both calcium channel blockers, being both inhibitors of a similar potential for reducing LDH activity (Figure 6(a)). Concerning “mitochondrial” enzymes (OCT and GDH), the inhibitory effect of calcium channel blockers on OCT release was practically absent in livers from PH rats, and in the case of that of GDH, the calcium channel blockers were ineffective in inhibiting its release, but diltiazem induced a discrete but significant increase of GDH release in PH rat livers (Figure 6(a)). In the absence of an imposed flow rate, as with liver slices obtained from PH rats, hepatic enzyme release was much lower than that recorded from the perfused organ, and addition of calcium barely increased the release of the enzymes tested (not shown). Addition of either verapamil or diltiazem was practically ineffective to modify the rate of enzymes released induced by the PH (Figure 6(b)).

### 3.7. Effects of Perfusing Isolated Livers through Portal versus Cava Veins on Hepatic Enzymes Release in Sham-Operated and PH Animals

**Figure 7:**
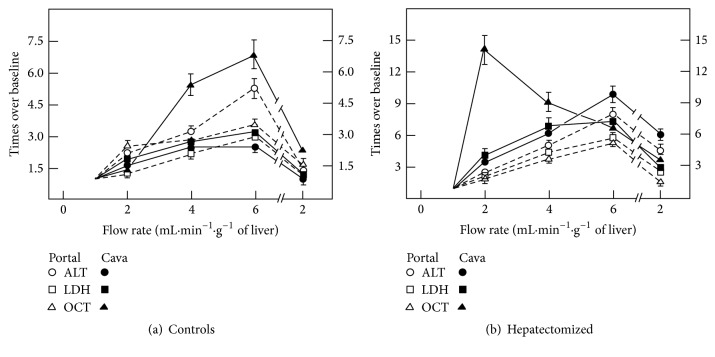
Effect of changing direction of flow rate on enzyme release from isolated livers of control and PH rats. Results of enzyme activities are expressed as mean ± SE (six independent determinations per experimental point) of times over a baseline flow rate of 1 mL·min^−1^·g^−1^ of liver for LDH, ALT, GDH, and OCT, in sham-operated and 70%-PH animals. Enzyme activities were measured in the perfusates from portal (empty) or cava (solid symbols) veins, under equal liver perfusion conditions to those shown in Figure 1.

The possible participation of endothelial cells in controlling liver enzyme release was assessed by changing the orientation of the flow rate. For this, we made a retrograde perfusion (through cava vein) in isolated livers from control and PH rats (Figure 7). In control animals, retrograde perfusion significantly diminished release of ALT and the release of mitochondrial enzymes tended to be enhanced only when compared with anterograde perfusion (Figure 7). On the contrary, the retrograde perfusion significantly promoted higher release of both OCT (Figure 7) and GDH (not shown in Figure 7).

## 4. Discussion

Based on previous communications from our group [10, 11, 21], we hypothesized that PH-induced selective enzyme release could be related to liver hemodynamic changes, which occur immediately after surgery. Based upon the results obtained, we demonstrated that liver enzyme release largely depends on extracellular calcium entry, probably mediated by stretch-sensitive calcium channels, as well as by increasing NO production. However, these effects were differentially observed when comparing liver enzymes from cytoplasmic or mitochondrial compartments. Moreover, a possible role for cell-mediated mechanotransduction in liver enzyme release was suggested by increasing shear stress, which also selectively affected the release of the enzymes tested. Therefore, the present study is an attempt to further extend the evidence of the stretch-sensitive calcium channels that participate in the liver enzyme release and to explore other intrinsic properties of the mechanotransduction events and the putative role of the endothelial glycocalyx.

Portal blood flow plays a pivotal role in the process of liver mass restoration after PH, since it results in a dramatic rise of the blood flow/liver mass ratio [31], and shear stress promotes liver NO release [32]. In this context, we have shown that flow-induced shear stress can control the amount of hepatic enzymes released into the bloodstream, through modifications in cell calcium mobilization and NO production [21]. Here, we show that liver enzymes release is largely controlled by mechanotransduction events acting on the hepatic endothelial cells and promoting changes in the intracellular distribution of the enzymes tested (Table 1).

As a first approach, we want to know whether the released enzymes are newly synthesized or there is a “pool” of these enzymes specifically determined to be delivered into the bloodstream. Results indicated that the increased flow rate-induced release of enzymes seemed to be independent of de novo protein synthesis, since serum enzyme activities were not significantly affected by treatment with CHM; however, CHM did affect in vitro liver enzyme release (Figure 1). This agrees with the stimulating effect of high amplitude-mechanical forces on liver protein synthesis and degradation [33], and shear stress could increase protein synthesis in the endothelial layer (i.e., glycocalyx mechanoreceptors). Indeed, we found that retrograde perfusion and degradation of the glycoproteins-containing carbohydrates in the mechanoreceptors modify liver enzyme release (Figures 2–4 and 7). Directionality or the vector component of the fluid flow affects biological responses, as in the formation of new vessels in the coronary sinus [34]. This matches the notion that modified anisotropy is relevant to physiological situations [35].

The possibility of the existence of pooled enzymes, independent of their metabolic functions, destined to be released to the extracellular medium, was supported by our experiments (Table 1). In fact, the LDH activity in the mitochondrial fraction was increased after surgery, which could be related to the named cell-to-cell lactate shuttle which can provide additional metabolic energy, through LDH activity located attached to the mitochondrial membrane [36]. Actually, increased mitochondrial associated-hexokinase-II activity prevents oxidative stress, reducing mitochondrial ROS generation in hepatocellular carcinoma cells [37]. Hence, intracellular distribution of enzyme activities could control cell processes such as proliferation and/or apoptotic death, as suggested for glycolytic enzymes [38]. Then, the relative nuclear enrichment of enzyme activities (predominantly “mitochondrial”) in liver samples from PH animals might have a role in the nuclear transcriptional activity triggering an effective cell cycle during liver cell proliferation.

It is now known that the shear stress at the edge of the endothelial surface layer is greatly attenuated by the extracellular matrix of proteoglycans and glycoproteins in the glycocalyx, with the result that fluid velocities, except near the edge of the layer, are vanishingly small [39]. Thus, the shear stress due to the fluid flow acting on the apical membrane of the endothelial cell itself is negligible. This paradoxical prediction has raised a fundamental question as to how hydrodynamic and mechanical forces, more generally, are transmitted across the structural components of the glycocalyx [40, 41]. Therefore, we also assessed the possibility that CHM was affecting the synthesis of protein components of the endothelium glycocalyx, participating in mechanotransduction events. The enzymatic removal of carbohydrate residues did affect differentially the release of cytoplasmic enzymes and that of mitochondrial enzymes, such as OCT and GDH activities (Figures 2–4). Glycocalyx hyaluronan and/or aggregating proteoglycans are involved in the mechanotransduction of shear stress and the production of NO in endothelial cells [29, 30, 42]. Heparanase pretreatment reduces the pressure-induced increase of lung endothelial conductivity, suggesting that cell surface heparan sulfates directly participate in mechanotransduction that results in NO/ROS production and increased permeability [30]. Integrity of the vascular endothelial cell glycocalyx regulates mediation of coronary flow on heart glycolytic flux [27]. Removal of chondroitin sulfate had no effect on enzyme release (not shown). This agrees with the report that depletion of heparan sulfate, hyaluronic acid, and sialic acid, but not chondroitin sulfate, blocked shear-induced NO production, as well as that of MDA that we found in the present study (Table 2). Therefore, the endothelial glycocalyx could control liver enzyme release, since endothelial cells sensing a positive shear stress aligned parallel to the direction of blood flow [43]. Molecular mechanisms of mechanotransduction remain unexplored, but growth factors and their signalling pathways could be playing a major role [44]. However, the molecule(s) responsible for the translation of biomechanical forces into biochemical signals (mechanotransduction) have not been identified as yet, but the glycocalyx has been added to the list of possible candidates [45]. In the same context, it is known that increased NO synthesis could be implicated in the pathogenesis of the hemodynamic disturbances frequently found in cirrhotic patients, through activation of an inducible isoform of nitric oxide synthase [46].

Stretching forces induce increases in intracellular calcium concentration, an effect inhibited by gadolinium, suggesting that this response must rely on the entry of extracellular calcium through the stretch-sensitive channels [47]. In this regard, mechanical stimulation determines the dynamics of current responses of dorsal root ganglion neurons, activating them as a function of membrane stretch [48]. Here, we have demonstrated that liver enzyme release is linked to extracellular calcium dynamics, mainly associated with mechanical forces as shear stress, since practically, in its absence (i.e., in liver slices, Figure 6), the calcium dependence for its intracellular entry was indeed abrogated. It has been reported that DNA synthesis is dependent on extracellular calcium in regenerating and nontransformed hepatocytes [49]. Moreover, we demonstrated that the calcium release channels (intracellular) are targets of mechanisms of metabolic control during the proliferative response following 70% PH and might be part of the modified intracellular Ca^2+^ dynamics during liver regeneration [50]. Therefore, we think that other functions of calcium dynamics might be the control of liver enzyme release, although the fate and function of the released enzymes are still unknown.

Mechanical force application can activate mechanosensitive ion channels and trigger calcium entry into cells and, consequently, modulate cytoskeleton structure or contractility [23]. Indeed, enzyme release depends on extracellular calcium entry, probably mediated through endothelial stretch-sensitive channels, exerting a differential effect on liver enzymes release [21]. Direct and specific stretch of *α*1 integrin elicits Cl^−^ channels functioning in ventricular myocytes, involving angiotensin II release, engagement of AT1 receptors, NADPH oxidase activation, and ROS production [51]. In this context, purinergic P2 and P1 receptors confer some of the KCNQ1 channel volume sensitivity in oocytes, although endogenous adenosine receptors and expressed P2Y2 receptors do so in the negative direction, as responses to cell volume changes [52]. Actually, adenosine restores desensitized angiotensin II-induced contractions in the renal arterioles via intracellular, receptor-independent mechanisms including the p38 mitogen-activated protein kinase (MAPK). This effect seems to involve an increased calcium sensitivity of the vascular contractile apparatus [53]. Adenosine has been pointed out as an important molecule involved in the mechanotransduction events [12] and when receptors, ion channels, and signalling molecules are administered [16, 54, 55], the endothelial glycocalyx components might be the “mechanotransducers” for fluid stress [20, 30]. We found that NO production is stimulated by increasing flow rate in isolated livers, an effect greatly exacerbated when perfusing organs from PH rats [21]. This agrees with the notion that all portal flow must go through the remaining vascular bed, increasing shear stress and promoting NO release [32, 56].

The universality of physical responses to stretch has been established, pointing out that cell stretch seems to provoke ongoing physical events in cell signalling; therefore, physical forces are essential in the process of mechanotransduction [57]. Moreover, cells on stiffer substrates have more organized cytoskeletons and stable focal adhesions [58]. Therefore, present data support the statement that hemodynamic changes induce mechanotransduction-mediated signals that impact the hepatocytes, responding with differential enzyme release into the bloodstream. In addition, we have recently demonstrated that in vitro modifications of the oxidant status affect differentially the release of liver enzymes, indicating that this release is a strictly controlled event and is not directly related to the onset of oxidant stress of the liver [59].

An interesting and recent study indicates that microRNA-21 (miR-21) could serve as an important regulator driving hepatocyte proliferation after PH in mice and the suppression of miR-21 revealed its impact in the balance of proproliferative effects with antiproliferative profibrogenic actions in regulating distinctive regenerative responses in normal or in disease conditions [60]. Moreover, fluctuations in shear stress contribute to miR-regulated differential gene expression in endothelial cells, maintaining vascular physiology. Particularly, changes in the expression profile of miR-21 and miR-92a by high shear stress are associated with an atherogenic protective function [61]. Therefore, it is not unlikely to assume the existence of connections or interactions between miRNAs expression and enzymes release in processes as cell proliferation and responsive to hemodynamic changes.

Under oxidant circumstances, intracellular homeostasis in the redox status is interrupted and induces liver cell damage. This results in apoptosis or necrosis, potentially contributing to the devastating injury and dysfunction of liver tissue, where it has been shown that the p47^phox^ subunit of NADPH oxidase complex participates actively in the liver damage. Here, TNF-*α* and IL-6 also would induce endoplasmic reticulum stress, which elicits the unfolded protein response, which occurs with evident release of liver enzymes in alcoholic and nonalcoholic liver diseases [59]. However, the latter that could be considered as the “canonical” molecular mechanism for enzyme release as a consequence of liver cell necrosis could be revised according to our new data. In fact, we propose that increased cytoplasmic/mitochondrial enzyme activity ratios are more linked to active proliferation of the liver parenchyma, which does not seem to be related to cell necrosis and coincides with the progression of PH-induced rat liver regeneration at the various stages of the cell cycle [21]. Therefore, the latter might have clear clinical consequences, mainly at diagnostic and patient's management level.

A question arises of whether the liver enzyme release into the bloodstream could have a physiological role. We really ignore the possible role of serum enzymes in the intermediary metabolism, as well as whether these actively catalytic proteins can indeed use substrates in the serum blood fraction. However, we have recent evidence that OCT, which is localized in the liver mitochondrial matrix, is largely released during rat liver regeneration, and, in this situation, extracellular and circulating hepatic OTC could be playing a different role, possibly functioning as a signalling molecule [62].

## 5. Conclusions

Although we are aware that some experimental approaches were performed with nonspecific pharmacological interventions, we think there is enough evidence that flow-induced shear stress is an important component in the underlying mechanisms that control the amount of serum activities of hepatic enzymes. We show that this physical force probably acting on both the endothelium and the extracellular matrix-hepatocytes interface could be an essential element to stimulate release of enzymes from the intact liver. Here, cell calcium mobilization largely dependent on its extracellular influx through stretch-sensitive calcium channels, as a main response of the influence exerted by hemodynamic changes, could play an important role, probably through differential modulation of still undefined biochemical pathways. Since a possible function of circulating levels of enzyme activities could pertain to a complex signalling system, its clinical determination could also reflect hemodynamic changes occurring in tissues subject to injury-repair episodes that underlie most of the human pathologies. This phenomenon is exacerbated in the PH-induced proliferating rat liver and the selectivity of the released enzymes could somehow be related to signalling pathways, which can be therefore useful in the interpretation of the elevation of serum enzyme activities. Moreover, these results open a new scenario for evaluating the possible clinical consequences that can occur in patients medicated with drugs impacting hemodynamic and/or systemic blood pressure, as those taking vasodilators and Ca^2+^ antagonists, in relation to the mechanotransduction-mediated release of liver enzymes.

## Figures and Tables

**Table 1:** Intracellular distribution of enzyme activities determined in livers from sham-operated and PH animals.

	LDH	ALT	AST	OCT	GDH
Cytosol (% of total activity)					
Sham	75 ± 5	73 ± 6	44 ± 5	8 ± 1	0.6 ± 0.1
PH, 12 h	53 ± 7^**∗**^	58 ± 12	32 ± 7	3 ± 0.4^**∗**^	0.6 ± 0.1
PH, 24 h	51 ± 4^**∗**^	76 ± 10	45 ± 7	7 ± 1	1.0 ± 0.2
PH, 48 h	57 ± 8	72 ± 10	39 ± 5	6 ± 1	0.7 ± 0.2
PH, 72 h	62 ± 12	68 ± 11	35 ± 6	5 ± 1	0.4 ± 0.1
Mitochondria (% of total activity)					
Sham	2.5 ± 0.4	3 ± 0.5	28 ± 4	71 ± 9	66 ± 6
PH, 12 h	18 ± 3^**∗**^	5 ± 0.7^**∗**^	46 ± 8^**∗**^	90 ± 4	78 ± 11
PH, 24 h	14 ± 2^**∗**^	3 ± 0.6	35 ± 5	64 ± 5	63 ± 4
PH, 48 h	9 ± 2^**∗**^	3 ± 0.6	36 ± 5	69 ± 6	69 ± 6
PH, 72 h	6 ± 0.8^**∗**^	2.5 ± 12	33 ± 4	72 ± 8	67 ± 9
Microsomes (% of total activity)					
Sham	16 ± 1	8 ± 1	10 ± 2	1.5 ± 0.4	15 ± 2
PH, 12 h	20 ± 3	8 ± 1	8 ± 1	1 ± 0.1	6 ± 2^**∗**^
PH, 24 h	28 ± 3^**∗**^	8 ± 1	11 ± 2	3.4 ± 0.6^**∗**^	18 ± 2
PH, 48 h	22 ± 3	10 ± 2	11 ± 2	2.4 ± 0.5	15 ± 3
PH, 72 h	21 ± 3	9 ± 2	12 ± 3	1.3 ± 0.2	13 ± 2
Plasma membranes (% of total activity)					
Sham	6 ± 1	10 ± 1	16 ± 3	19 ± 3	17 ± 3
PH, 12 h	9 ± 2	15 ± 2^**∗**^	10 ± 2	6 ± 1^**∗**^	14 ± 3
PH, 24 h	7 ± 1	9 ± 1	8 ± 2^**∗**^	25 ± 4	15 ± 2
PH, 48 h	9 ± 2	13 ± 2	12 ± 2	23 ± 3	19 ± 3
PH, 72 h	11 ± 2^**∗**^	15 ± 2^**∗**^	18 ± 3	21 ± 4	18 ± 2
Nuclei (% of total activity)					
Sham	0.14 ± 0.02	6 ± 1	2.0 ± 0.3	0.1 ± 0.03	1.5 ± 0.2
PH, 12 h	0.07 ± 0.02	12 ± 2^**∗**^	3.3 ± 0.7	0.2 ± 0.04	1.1 ± 0.2
PH, 24 h	0.23 ± 0.04	4 ± 1	1.5 ± 0.3	0.8 ± 0.10^**∗**^	2.1 ± 0.2^**∗**^
PH, 48 h	0.18 ± 0.03	6 ± 1	1.8 ± 0.3	0.3 ± 0.06^**∗**^	1.8 ± 0.3
PH, 72 h	0.11 ± 0.02	7 ± 1	2.3 ± 0.3	0.2 ± 0.02	1.5 ± 0.2

Percentages for enzyme activities are the mean ± SE of seven individual observations per experimental point for sham-operated control of partial hepatectomized (PH) rats. Statistical significance: ^*∗*^*p* < 0.01 against the control group.

**Table 2:** Effect of flow rate and of glycocalyx modifiers in the content of NO and LP by-products (MDA) in perfusates from livers of sham-operated and PH animals.

Flow rate	mL·min^−1^·g^−1^ of liver		
	At 2	At 4	At 6
NO production (pmoles·min^−1^·g^−1^ of liver)			
Sham + none	155 ± 19	235 ± 35	300 ± 51
Sham + hyaluronidase	132 ± 15	155 ± 22^*∗*^	160 ± 25^*∗*^
Sham + heparanase	162 ± 21	176 ± 35	142 ± 28^*∗*^
Sham + concanavalin A	125 ± 16	136 ± 23^*∗*^	137 ± 24^*∗*^
NO production (pmoles·min^−1^·g^−1^ of liver)			
PH + none	257 ± 40^*∗*^	519 ± 73^*∗*^	353 ± 60
PH + hyaluronidase	151 ± 23^*∗∗*^	185 ± 26^*∗∗*^	183 ± 26^*∗∗*^
PH + heparanase	233 ± 42	389 ± 52^*∗*^	198 ± 39^*∗∗*^
PH + concanavalin A	215 ± 38	290 ± 53^*∗∗*^	207 ± 35^*∗∗*^
MDA production (nmoles·min^−1^·g^−1^ of liver)			
Sham + none	14 ± 2	19 ± 3	32 ± 5
Sham + hyaluronidase	12 ± 2	14 ± 4	20 ± 4
Sham + heparanase	10 ± 2	15 ± 3	16 ± 4^*∗*^
Sham + concanavalin A	11 ± 2	13 ± 3	18 ± 3^*∗*^
MDA production (nmoles·min^−1^·g^−1^ of liver)			
PH + none	23 ± 3^*∗*^	35 ± 4^*∗*^	65 ± 8
PH + hyaluronidase	16 ± 3	15 ± 4^*∗∗*^	40 ± 7^*∗∗*^
PH + heparanase	16 ± 3	22 ± 3^*∗∗*^	31 ± 5^*∗∗*^
PH + concanavalin A	19 ± 3	24 ± 4	45 ± 7^*∗*^

The results are the mean ± SE of five individual observations per experimental point for sham-operated control of partial hepatectomized (PH) rats, for nitric oxide (NO) and malondialdehyde (MDA) production. Statistical significance: ^*∗*^*p* < 0.01 against the control group, ^*∗∗*^*p* < 0.01 versus the PH group.
